# Backward Estimation of Exposure to Organochlorines using Repeated Measurements

**Published:** 2004-07

**Authors:** Wilfried Karmaus

Karmaus et al. detected errors in their article “Backward Estimation of Exposure to Organochlorines using Repeated Measurements” [ Environ Health Perspect 112:710–716 (2004)]. In Table 1, values in the equations for the proposed regression model were incorrect. The correct equations for Table 1 are as follows:

For estimation of 1970s values from 1980s values,


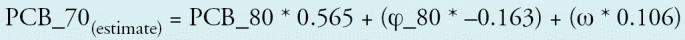


For estimation of 1980s values from 1990s values


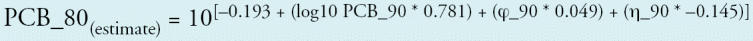


Also, in Figure 3, there should not be a measurement for the year 2010. The authors appologize for the errors.

